# A computational study of mechanical properties of collagen-based bio-composites

**DOI:** 10.1080/23335432.2020.1812428

**Published:** 2020-09-02

**Authors:** Marco Fielder, Arun K. Nair

**Affiliations:** a Multiscale Materials Modeling Lab, Department of Mechanical Engineering, University of Arkansas, Fayetteville, AR, USA; b Institute for Nanoscience and Engineering, University of Arkansas, Fayetteville, AR, USA

**Keywords:** Bio-composites, Molecular dynamics, Nanoscale deformation mechanisims

## Abstract

Studying changes in collagen deformation behavior at the nanoscale due to variations in mineralization and hydration is important for characterizing and developing collagen-based bio-composites. Recent studies also find that carbon nanotubes (CNTs) show promise as a reinforcing material for collagenous bio-composites. Currently, the effects of variation in mineral, water, and CNT content on collagen gap and overlap region mechanics during compression is unexplored.

We use molecular dynamics simulations to investigate how variations in mineral, water, and CNT contents of collagen bio-composites in compression change their deformation behavior and thermal properties. Results indicate that variations in mineral and water content affect the collagen structure due to expansion or contraction of the gap and overlap regions. The deformation mechanisms of the gap and overlap regions also change. The presence of CNTs in non-mineralized collagen reduces the deformation of the gap region and increases the bio-composite elastic modulus to ranges comparable to mineralized collagen. The collagen/CNT bio-composites are also determined to have a higher specific heat than the studied mineralized collagen bio-composites, making them more likely to be resistant to thermal damage that could occur during implantation or functional use of a collagen collagen/CNT bio-composite biomaterial.

## 1. Introduction

Collagen is a fundamental part of biological tissues such as bone, tendon, muscle, and skin (Fratzl 2008). A hierarchical structure is a primary characteristic of collagen-based bio-composites (Currey 2002; Fratzl 2008). At the nanoscale, collagen in bone is mainly bundles of fibrils embedded in an extrafibrillar mineral matrix. The fibrils are a composite of primarily type I collagen protein, intrafibrillar mineral, and water. The collagen molecules are arranged in a periodic triclinic structure (Wess et al. 1995; Orgel et al. 2006), forming regions where there are gaps and overlaps between collagen molecules. The combined length of a single gap and overlap region forms what is known as *D*-banding, where *D* is approximately 67 nm (Nikolov and Raabe 2008; Streeter and de Leeuw 2010). The brittle mineral phase is primarily hydroxyapatite (HAP), and experiments have shown that fibril mineralization initially begins in the gap region (Nudelman et al. 2010; Wang et al. 2012). Water fills the remaining fibril voids and can be either mobile water typically found in pore channels, or structural water found between collagen and mineral molecules. (Timmins and Wall 1977; Nyman et al. 2006; Zhang et al. 2007; Gul-E-Noor et al. 2015).

Inspired by the nanoscale structure and composition of bone, this study investigates collagen-based bio-composites to aid the development of biocompatible materials. Studying the nanoscale structure and mechanics of collagenous tissues and composites is important because their macroscale properties can change depending on many factors. For example, mineral and water content in bone have been found to decrease as a person gets older, making them more brittle (Mueller et al. 1966; Rauch and Glorieux 2004; Saxon et al. 2014). These changes in properties begin with nanoscale changes in bone structure and composition (Hamed et al. 2010; Pradhan et al. 2011; Hamed and Jasiuk 2013; Gul-E-Noor et al. 2015; Depalle et al. 2016).

The effects of the nanoscale mechanics and structure of collagenous tissues on their ultrastructural mechanical properties and the underlying mechanisms responsible for these effects have been studied using experimental techniques such as x-ray diffraction (Sasaki and Odajima 1996), atomic force microscopy (Eppell et al. 2006; van der Rijt et al. 2006; Minary-Jolandan and Yu 2009), and nuclear magnetic resonance spectroscopy (Gul-E-Noor et al. 2015). Computational methods such as molecular dynamics simulations (Zhang et al. 2007; Streeter and de Leeuw 2010; Tang et al. 2010; Gautieri et al. 2011; Nair et al. 2013; Depalle et al. 2016), finite element analysis (Thomopoulos et al. 2006; Hamed and Jasiuk 2013; Ahsan 2017; Lin et al. 2017), and ab initio methods (Dubey and Tomar 2013) have also been used to study the mechanical properties of collagenous tissues.

Studying the thermal properties of collagen-based bio-composite scaffolds is important because a high specific heat capacity and thermal conductivity may prevent damage if the functional use of the bio-composite (or the placement of it *in vivo*) produces a lot of heat. Experiments by Holmes et al. find that telopeptide cross-link cleavage alters the flow of heat and structure of collagen (Holmes et al. 2017). Qu and Tomar used molecular dynamics simulations to study the thermal properties of collagen-hydroxyapatite nanocomposites and found that the weight fractions, material geometry, and strain all affect the bio-composite’s thermal properties (Qu and Tomar 2015).

**Figure 1.**
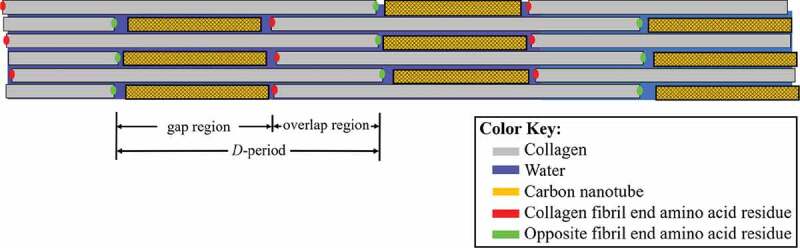
A collagen fibril visualized as a schematic. The collagen molecule end residues are represented by green and red dots to highlight the ends of the gap and overlap regions

Carbon nanotubes (CNTs) are being studied as a material to strengthen collagen-based bio-composites (MacDonald et al. 2005; Zanello et al. 2006; Meng et al. 2009; Hirata et al. 2011; Amirian et al. 2012; Jing et al. 2017; Silva et al. 2017; Tanaka et al. 2017). Studies investigating the structure of collagen/CNT bio-composites observed the nanotubes primarily in the collagen gap region. A schematic of the structure of a collagen fibril with CNTs can be seen in Figure 1, with the CNTs between collagen molecules in the gap region. Carbon nanotubes (CNTs) have excellent thermal and electrical conductivity (Balandin, 2011; Charlier et al. 1996), effective bio-molecular adsorption properties (Kang et al. 2008), and a relatively high elastic modulus of about 1 TPa (Zhang et al. 2002). This makes CNTs practical as a stand-alone material or for use in composites in applications that require high stiffness.

Previous studies have investigated the nano-mechanics of collagenous bio-composites as a singular function of either water content or mineral content. However, both mineral and water content in collagen can vary simultaneously and it has yet to be investigated how this affects the mechanical and thermal properties of collagenous bio-composites. Previous studies have also focused on the structure and biological viability of collagen/CNT bio-composites, but the nanoscale mechanics and thermal properties of collagen/CNT bio-composites and its comparison to mineralized collagen is still unexplored. We investigate how the mechanical and thermal properties of collagen-based bio-composites, simulated with molecular dynamics, change as a function of both mineral and water content. We also compare the elastic modulus values of the bio-composite to established theoretical models. Finally, we aim to determine if collagen/CNT bio-composites can mimic the structure, mechanics, and thermal properties of mineralized collagen bio-composites.

## 2. Materials and methods

**Figure 2.**
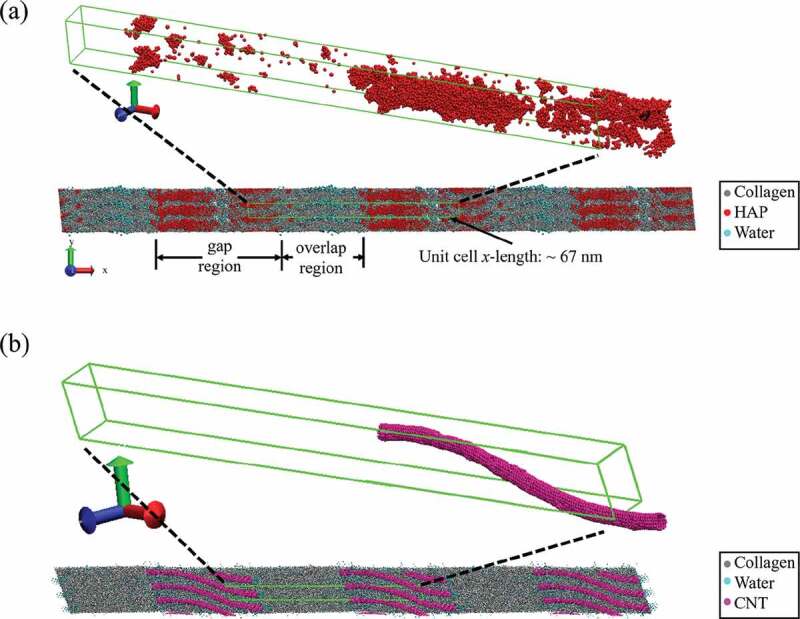
(a) The structure and periodic unit cell of a collagenous bio-composite with 40 wt% mineral and 3.3 wt% water. (b) The structure and periodic unit cell of a collagenous bio-composite with 10 wt% CNT and 20 wt% water

Studying the nanoscale mechanics of bio-composites can be challenging using experimental techniques. One method for studying their nanoscale mechanics is through computational methods such as molecular dynamics (MD). In this study, MD simulations are performed using the Large-scale Atomic/Molecular Massively Parallel Simulator (LAMMPS) program (Plimpton 1995). Details on the development of the bio-composites are available in the S1 section of the supplementary material. The bio-composites are mineralized at 0, 20, and 40 wt% to model varying degrees of mineralization. The bio-composites are hydrated at 0, 2, 4, 10, or 20 wt% water. Figure 2(a) shows the equilibrated bio-composite gap and overlap regions, and the model unit cell whose length along the *x*-axis corresponds to the *D*-period length of 67 nm. Additionally, the models have periodic boundary conditions in the *x, y*, and *z* directions to model larger conformations of fibrils. A zoomed-in view of the unit cell shows the distribution of mineral primarily in the gap region.

Details of the development of the CNT models for the collagen/CNT bio-composites are available in the S2 and S3 sections of the supplementary material. We vary the water content from 10 wt% to 20 wt% for a bio-composite with 10 wt% single-walled carbon nanotubes (SWCNTs). Higher water contents of 10–20 wt% are chosen for the collagen/CNT composites in order to compare their mechanical properties to the non-mineralized collagen composites with the water content of healthy fibrils (10–20 wt%), while the water contents of the mineralized composites were varied until they reached modulus values comparable to the collagen/CNT composites with 10–20 wt% water content. We also compare collagenous bio-composite with 7 wt% SWCNTs with 11 wt% multi-walled carbon nanotubes (MWCNTs). Figure 2(b) shows an equilibrated bio-composite with 10 wt% SWCNT and 20 wt% water. A zoomed-in view of the unit cell shows a single SWCNT per unit cell. In agreement with experiment, the SWCNT bends slightly during equilibration to fit between collagen molecules in the gap region (MacDonald et al. 2005; Tan et al. 2010). Table 1 shows the material contents for all the bio-composites in our study, as well as the total number of atoms in each model.

**Table 1.** Bio-composite material contents

Mineral content (wt%)	CNT content (wt%)	Water content (wt%)	Number of Atoms
0	0 (SWCNT)	2	39738
0	0 (SWCNT)	4	40749
20	0 (SWCNT)	2	43266
20	0 (SWCNT)	4	45063
40	0 (SWCNT)	2	49448
40	0 (SWCNT)	4	50405
0	4 (SWCNT)	20	52724
0	7 (SWCNT)	20	54481
0	10 (SWCNT)	10	56127
0	10 (SWCNT)	20	48693
0	11(MWCNT)	20	57161

The method for applying quasi-static stress is the same as used in a previous study (Fielder and Nair 2018), and further details are available in the S1 section of the supplementary material. In short, the unit cell dimensions are allowed to change until the pressure (which corresponds to the stress) of the unit cell in the *x*-direction reaches the target stress value. The compressive stress is plotted vs. the linear strain, the Young’s modulus is plotted vs. the water content, and we plot the gap/overlap ratio vs. applied stress. The gap and overlap region lengths are determined by the measuring distance between the terminal amino acid residues of the collagen molecules, which corresponds to the beginning and end of the gap and overlap regions. This can be seen in the schematic of Figure 1 with the red and green points showing the distances between the gap and overlap regions. The thermal properties of the bio-composites are determined by the specific heat capacity, whose method of calculation can be found in section S1 of the supplementary material. Briefly stated, based on the total energy, pressure, and volume of the simulations, the enthalpy of the system can be determined. Subsequently, the specific heat capacity can be calculated utilizing the ideal gas constant and the enthalpy, mass, and temperature of the system.

## 3. Results and discussion

### 3.1 Stress vs. Strain

**Figure 3.**
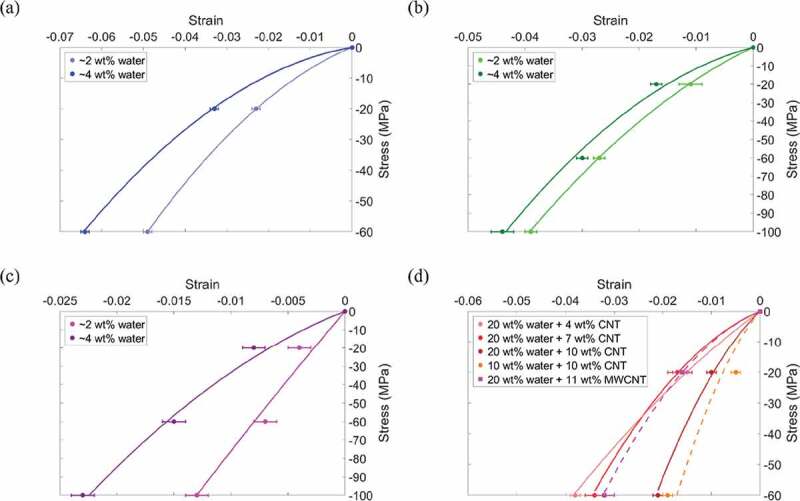
Compressive stress vs. strain of collagenous bio-composites. Bio-composites without CNTs and with mineral contents of (a) 0 wt%, (b) 20 wt%, and (c) 40 wt%. (d) Bio-composites containing CNTs but no mineral

The compressive stress vs. strain is plotted in Figure 3 for all samples. We find that as the water content increases the strain increases, and as the mineral content increases from 0 to 20 to 40 wt% (Figure 3(a–c) respectively), the strain decreases, and the stress–strain relation becomes more linear. Next, we compare the effect of an increase in water content from 2 to 4 wt% at an applied stress of 60 MPa and find that at 0, 20, and 40 wt% mineral there is 30%, 10% and 114% increase in strain, respectively. Comparing the bio-composites with 2 wt% water at 60 MPa applied stress, an increase in mineral content from 0 wt% to 20 wt% results in 44% decrease in strain. A further increase in mineral content from 20 wt% to 40 wt% results in 74% decrease in strain.

The stress vs. strain for collagen/CNT bio-composites is shown in Figure 3(d). At 60 MPa stress, an increase in SWCNT content from 4 wt% to 10 wt% results in 45% decrease in strain. The bio-composites with CNTs have strain values comparable to those of mineralized bio-composites without CNTs. We see at 60 MPa applied stress that the maximum strain for bio-composites with CNTs is 3.8% while for bio-composites with 20 wt% mineral the maximum strain is 4.4%. At 60 MPa stress, the bio-composites with 40 wt% mineral still had the overall minimum strain at 0.7%, however, the weight fraction of CNTs reinforcing the bio-composite is lower than the weight fraction of mineral needed to reach a similar strain value.

### 3.2 Elastic modulus

**Figure 4.**
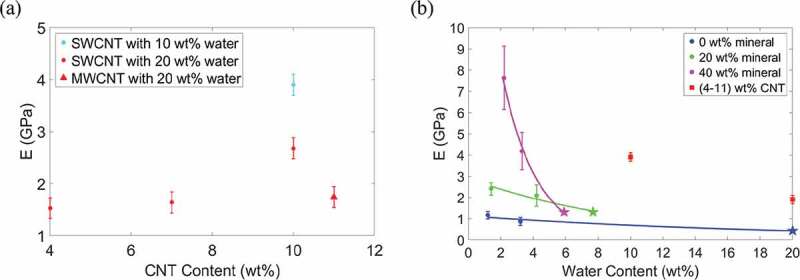
(a) Compressive Young’s modulus vs. bio-composite CNT content. (b) Compressive Young’s modulus vs. bio-composite water content. The modulus values determined by Nair et al. (Nair et al. 2014) are plotted as pentagram-shaped data points

The Young’s modulus values for the bio-composites are plotted in Figure 4. The modulus values determined in a previous study by Nair et al. (Nair et al. 2014) using a similar model are also shown in Figure 4 for comparison and are represented by pentagram-shaped data points. We compare the effect of an increase in water content from 2 to 4 wt% on the bio-composites’ modulus values. Overall, as the water content increases, the modulus decreases. The trend is nonlinear to account for a saturation point where an increase in water content will have a negligible effect on the composite modulus. For compositions with 0, 20, and 40 wt% mineral, an increase in water content results in decreases in modulus by 26%, 13%, and 45%, respectively. We see that as the mineral content increases from 0 to 20 wt%, and subsequently from 20 to 40 wt%, the magnitude of the decrease in modulus increases. This is likely as the water is having a more significant effect on the dominant deformation mechanism of mineralized bio-composites (shearing of collagen between mineral phases) than that of the non-mineralized bio-composites (collagen bending). Evidence of the deformation mechanisms is discussed further in section 3.3.

An increase in SWCNT concentration from 0 to 10 wt% results in an increase in modulus. Due to small variation, the average modulus of bio-composites with 20 wt% water in Figure 4(a) is plotted in Figure 4(b) as a single value. In Figure 4(b), the modulus values for the collagen/CNT bio-composites are comparable to those of the mineralized bio-composites, ranging from approximately 1.4 to 4 GPa. As the bio-composite mineral content increases, the modulus increases. However, the presence of water reduces the degree to which mineralization changes the modulus. For example, in Figure 4(b) at 2 wt% water, for an increase in mineral content from 0 to 40 wt% the modulus increases from 1.16 to 7.63 GPa. At 4 wt% water, an increase in mineral content from 0 to 40 wt% results in an increase in modulus from 0.86 to 4.18 GPa.

We compare the bio-composite compressive modulus values from Figure 4 to fibril compressive modulus values determined in other studies, which is shown in Table 2. Studies of highly mineralized fibrils that are dry or fully hydrated are compared to the 40 wt% mineral models in Figure 4, with 2 and 6 wt% water, respectively. Studies of non-mineralized fibrils that are dry or fully hydrated are compared to the 0 wt% mineral models in Figure 4, with 2 and 20 wt% water, respectively. The bio-composite modulus values for this study are in good agreement with the modulus values of the other studies in Table 2. The results of this study also give insight into the modulus values of partially mineralized or hydrated collagenous bio-composites. This is crucial since mineral and water content may vary due to external factors, or the mineral and water distribution within the bio-composite may not be homogeneous. This can be seen in Table 2 where, for the experimental values from other studies, there is often a significant variation in the compressive modulus values at the same mineral and water contents. The variation in compressive modulus is likely due to factors not considered in these experiments such as the amount of mineral substitution, the amount of mobile vs. bound water, and the presence of defects or non-collagenous proteins, all of which can change the bio-composite modulus. It should also be noted when comparing to the MD study by Dubey and Tomar (2009), that their mineralized fibril models were created with idealized geometric structure, which accounts for their variations in modulus values.

**Table 2.** Compressive Young’s moduli obtained by other studies of collagen fibrils

Method of Testing	Mineral Content	Water Content	Young’s modulus (GPa)
MD (this study)	0 wt%	~2 wt%	1.16
MD (Nair et al. 2014)	0 wt%	20 wt%	0.43
MD (this study)	40 wt%	~2 wt%	7.36
MD (Nair et al. 2014)	40 wt%	6 wt%	1.31
AFM nanoindentation (Grant et al. 2008)	0 wt%	0 wt%	1.9 ± 0.5
AFM nanoindentation (Grant et al. 2008)	0 wt%	fully hydrated	1.3 ± 0.1
AFM nanoindentation (Kemp et al. 2012)	0 wt%	0 wt%	1.26 ± 0.354
AFM nanoindentation (Kemp et al. 2012)	0 wt%	fully hydrated	0.03 ± 0.01
AFM nanoindentation (Minary-Jolandan and Yu 2009)	0 wt%	0 wt%	1.2 to 2.2
AFM nanoindentation (Grant et al. 2008)	0 wt%	0 wt%	1.9 ± 0.5
MD (this study)	40 wt%	~2 wt%	7.36
MD (Nair et al. 2014)	40 wt%	6 wt%	1.31
AFM nanoindentation (Lefèvre et al. 2013)	highly mineralized	0 wt%	13.87 ± 8.24
AFM nanoindentation (Lefèvre et al. 2013)	highly mineralized	fully hydrated	0.003 ± 0.001
MD (Dubey and Tomar 2009)	highly mineralized	0 wt%	7 to 11
MD (Dubey and Tomar 2009)	highly mineralized	fully hydrated	4 to 8
Synchrotron x-ray scattering/backscattered electron imaging (Karunaratne et al. 2012)	60 wt%	partially hydrated	6 to 10

**Figure 5.**
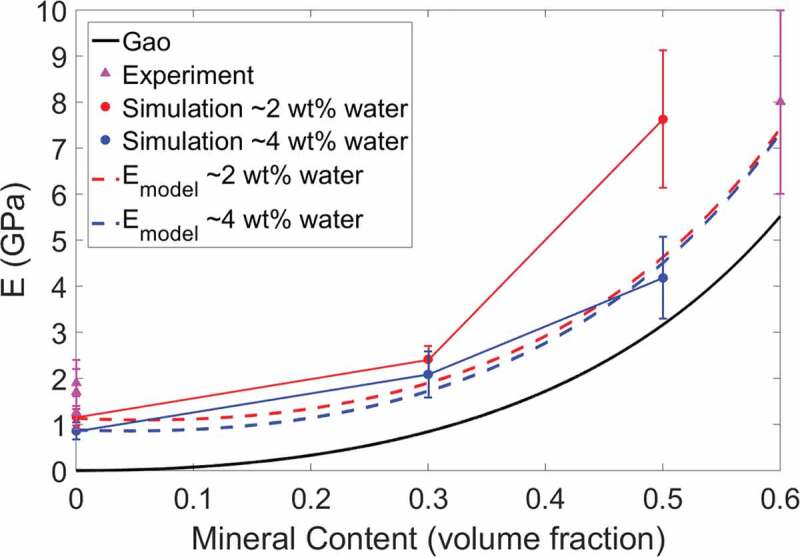
Compressive Young’s modulus vs. bio-composite mineral content of experimental, simulation, and predictive model results. The MD simulation compressive modulus is for bio-composites with water contents of 2 wt% (red) and 4 wt% (blue)

In Figure 5, the modulus values from this study are compared to those found using the Gao model (Ji et al. 2004) and the modified Padawer and Beecher model (Padawer and Beecher 1970). The formulation of these models are available in the S4 section of the supplementary material. The experimental values in Table 2 are also plotted in Figure 5 for comparison. As Figure 5 shows, the Young’s modulus results from this MD study agree well with the experimental modulus values, as they are within the error bars, and follow a similar trend as the Gao model. However, it should be noted that at low mineral volume fractions the Gao model effective modulus reduces to zero and does not account for the effect of the remaining collagen and water on the bio-composite modulus.

The formulated model for the bio-composites (based on the Padawer and Beecher model) agrees well with the experimental results from other studies in Figure 5. Our bio-composite model also agrees well with the simulation results, although at high mineral volume fractions the formulated model for bio-composites with 2 wt% water predicts modulus values lower than those from our MD simulations. This is likely because at higher volume fractions of mineral content, the models do not account for extrafibrillar mineralization, where mineral is also located between individual collagen fibrils, which also affects the deformation behavior. While beyond the scope of this paper, further investigation could incorporate the effect of extrafibrillar mineralization into bio-composite model. Linear regression analysis for the bio-composites determines that the linear relation between the formulated bio-composite model and the simulation results is significant and the model can accurately predict the modulus of the bio-composite (see section S4 of the supplementary material).

### 3.3 Gap/Overlap ratio and deformation behavior

**Figure 6.**
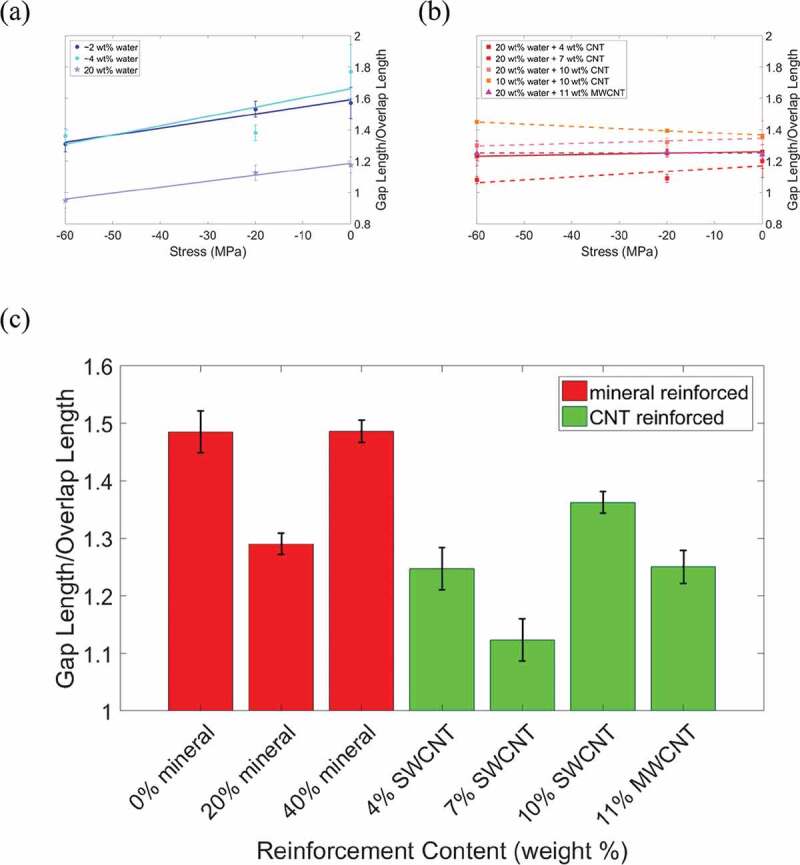
Gap/overlap ratio vs. compressive stress for (a) non-mineralized bio-composites, and (b) collagen/CNT bio-composites. (c) Bar graph of the average gap/overlap ratio for each type of bio-composite reinforcement

**Figure 7.**
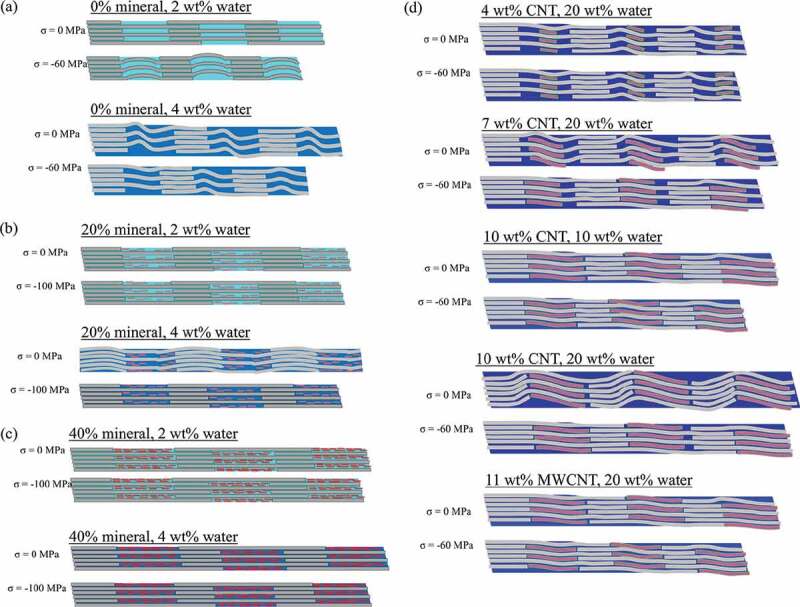
Schematics of the atomic trajectories showing the length of the bio-composite gap and overlap regions under compressive stresses (*σ*) ranging from 0 MPa and 100 MPa for bio-composites with (a) 0 wt% mineral, (b) 20 wt% mineral, (c) 40 wt% mineral, (d) collagen/CNT bio-composites

Next, we analyze the gap/overlap ratio (shown in Figure 6) and atomic trajectories to determine the deformation mechanisms (shown in the schematics of the atomic trajectories in Figure 7). At 0 wt% mineral and under no stress, an increase in water content from 2 wt% to 4 wt% results in a 13% increase in the gap/overlap ratio, as seen in Figure 6(a). This is because at low water contents the water primarily occupies gap region voids. As the water content increases, the gap region length expands while the overlap region length remains approximately the same, as seen in Figure 7(a). As the water content increases from 4 wt% to 20 wt% the water hydrates both the gap and overlap regions, which leads to expansion in both regions and a decrease in the gap/overlap ratio, as seen in Figure 6(a). Additionally, as the stress increases the gap/overlap ratios in Figure 6(a) decrease approximately linearly, demonstrating that the gap region deforms more than the overlap region under compressive stress.

In Figure 7(a), at 0 wt% mineral and 2 wt% water, the increase in stress results in compression/bending of the collagen molecules in the gap region. As the water content increases to 4 wt% and under no stress, the water causes slight folding of collagen in the gap region. As the applied stress increases on the bio-composite with 4 wt% water, the overall gap length decreases, but the collagen molecules also straighten out, which is characteristic of shearing.

The gap and overlap regions’ deformation behavior for bio-composites with 20 wt% mineral is shown in Figure 7(b). At 2 wt% water, the water is primarily in the overlap region voids due to the presence of mineral in the gap region. The gap/overlap ratio increases with increasing stress because the water results in larger compression of the overlap region, while mineral in the gap region resists compression, although this increase in gap/overlap ratio is relatively small. As the water content increases from 2 to 4 wt%, the gap/overlap ratio increases with no applied stress. This is because at 4 wt% water content the water distributes evenly throughout the bio-composite and causes gap region expansion. As stress is applied to the bio-composite with 4 wt% water, the gap/overlap ratio remains approximately constant at 1.3. The results in Figure 6(c) are also within the range of values determined in a previous study by Nair et al. (Nair et al. 2014). An increase in water content from 4 wt% to 8 wt% causes the gap/overlap ratio to decrease, since the overlap region expands due to hydration while the mineral in the gap region resists expansion. This agrees with experiments that found compressive pre-strain in the mineralized portions of hydrated collagenous bio-composites (Samuel et al. 2016). An increase in water content also led to an increase in the *D*-period of the simulated bio-composites. This agrees with the results of the previously mentioned experiment (Samuel et al. 2016), which also observed an increase in the *D*-period of mineralized fibrils as the water content increased.

For the 40 wt% mineralized bio-composites, at 2 wt% hydration the water is already uniformly distributed throughout the bio-composite. In addition, as the stress increases on the 40 wt% mineralized bio-composite samples, the gap/overlap ratio remains approximately constant, and while collagen bending due to hydration is observed, the amount of collagen bending is not significant relative to the total bio-composite size as shown in Figure 7(c). For the mineralized bio-composites under no stress an increase in water content from 2 to 4 wt% causes bending/folding of the collagen molecules in the overlap region, while the collagen in the gap region does not bend due to the minerals’ resistance to deformation, as shown in Figure 7(b). For both water contents, the increase in stress results in apparent shearing of the collagen molecules. We observe this by the straightening of the collagen molecules, particularly in the overlap region of the bio-composite with 4 wt% water.

The gap/overlap ratios for bio-composites with 40 wt% mineral is presented in Figure 7(c). The results at 2 wt% water and 4 wt% water are in good agreement with the results from a previous study of a fibril with 6 wt% water in (Nair et al. 2014), with a gap/overlap ratio between 1.4 and 1.6. Even at 2 wt% water, the water distributes uniformly throughout the bio-composite. As the water content increases the gap/overlap ratio decreases, since water causes an expansion of the overlap region, while the mineral in the gap region resists expansion of the gap region. Additionally, we again observe an increase in the *D*-period length with an increase in water content. As the stress increases, the gap/overlap ratio stayed approximately constant.

Next, we analyze the deformation mechanisms of the collagen/CNT bio-composites. Comparing Figure 6(a,b), for 0 wt% mineral and at no applied stress an increase in CNT content from 0 wt% to 4 wt% increases the gap/overlap ratio by 5%. This is because the CNTs cause the gap region to expand while the overlap region length remains approximately constant. As the CNT concentration increases to 7 wt%, the gap/overlap ratio decreases (Figure 6(b)). This is because, while the CNTs cause expansion of the gap region, they also displace water from the gap region to the overlap regions, which causes the overlap region to expand. At a CNT content of 4 wt%, the CNTs also displace water, but the CNTs only take up 28% of the gap region volume and the displaced water remains primarily in the gap region. At a CNT content of 7 wt%, CNTs take up 56% of the gap region volume so a significant amount of the water is displaced to the overlap region.

For an increase in CNT concentration (see Figure 6(b)) to 10 wt%, there is 11% increase in the gap/overlap ratio. At 10 wt% CNT, the CNTs occupy 83% of the gap region volume, expanding the gap region more than the overlap region expansion (which is displaced by water). Overall, as the stress increases, the gap/overlap ratio decreases. Although this shows the gap region deforms more than the overlap region, as stress increases, the decrease in gap/overlap ratio for bio-composites with CNTs is smaller than for bio-composites without CNTs. We next compare the bio-composites with 7 wt% CNTs (single-walled) and 11 wt% MWCNTs, as both cases contain 20 wt% water and 20 nm long CNTs. At no applied stress, the gap/overlap ratio of the bio-composites with MWCNTs is lower than those with SWCNTs because the larger MWCNT volume displaces additional water from the gap region. As stress increases, the gap/overlap ratio of the bio-composite with 7 wt% SWCNT decreases, while for the bio-composite with 11 wt% MWCNTs stays approximately constant. This demonstrates that the MWCNTs reduce the deformation of the gap region compared to the bio-composite with SWCNTs. At no applied stress (see Figure 7(d)) water causes folding of the collagen in the gap region. As the CNT concentration increases, this folding is reduced. As stress increases, although the overall bio-composite length decreases, the collagen molecules undergo straightening, a characteristic of shearing.

**Figure 8.**
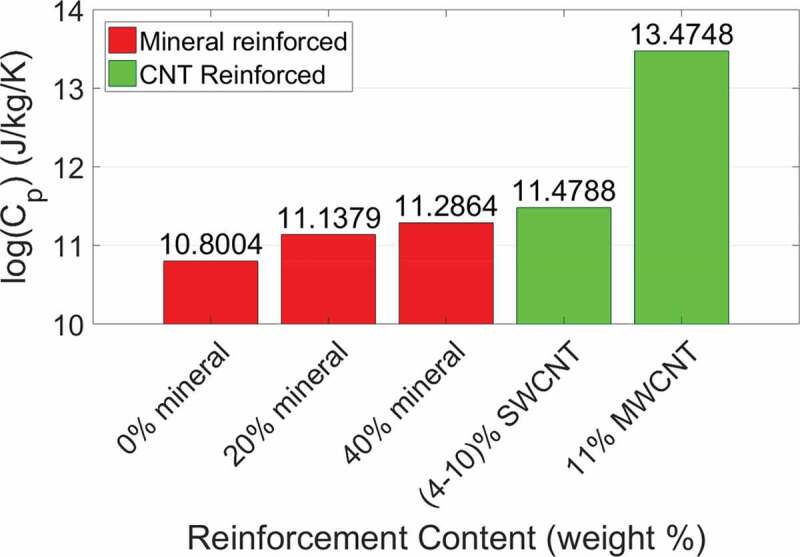
Bar graph of the logarithm of heat capacity *C_P_* for each type of bio-composite reinforcement

We compare the specific heat capacity for each of our models to the specific heat capacity values determined by Qu and Tomar (Qu and Tomar 2015) in Table 3. We find that the heat capacities for our models were an order of magnitude larger than those determined by Qu and Tomar. This is expected since heat capacity is dependent on the material mass, and the masses of the bio-composites in this study are an order of magnitude larger than the bio-composites studied by Qu and Tomar. For easier comparison we plot the logarithm of the bio-composite heat capacities in Figure 8. We see that an increase in mineral content increases the heat capacity due to an increase in the model mass, as observed by Qu and Tomar. A non-mineralized collagenous bio-composite with 4–10 wt% SWCNT has a 19% difference in heat capacity from a 40 wt% mineralized bio-composite, while a collagenous bio-composite with 11 wt% MWCNT has a 196% difference from a 40 wt% mineralized bio-composite. The heat capacity of CNTs and HAP are both in the range of 700 to 1000 Jkg^−1^K^−1^ (Pradhan et al. 2009; Qu and Tomar 2015). However, the collagen/MWCNT model has a significantly larger specific heat capacity. This is likely due to differences in electronic structure and phonon density of states between SWCNTs and MWCNTs as attributed in a study by Boldor et al. that found MWCNTs have a higher heat absorbance than graphite (Boldor et al. 2008).

**Table 3.** Average values of the bio-composites’ heat capacities

Bio-Composite Model	Heat Capacity *C_P_* (Jkg^−1^K^−1^)
0 wt% mineral reinforcement	49040 ± 4194
20 wt% mineral reinforcement	68728 ± 20154
40 wt% mineral reinforcement	79727 ± 13213
(4–10) wt% SWCNT reinforcement	96643 ± 65642
11 wt% MWCNT reinforcement	7112337 ± 73723
Collagen-HAP composite (Qu and Tomar 2015)	≈20000

## 4. Conclusions

We developed models of collagenous bio-composites with varying mineral, water, and CNT content and tested them under compressive loading using molecular dynamics to determine the viability of CNTs as a substitute for mineral in collagenous bio-composites. In addition, we studied the deformation behavior of the gap and overlap regions of the bio-composites and calculated their heat capacities. An increase in the mineral content increased the stiffness of the bio-composites and reduced the change in elastic modulus caused by the inclusion of water. The inclusion of CNTs in non-mineralized bio-composites also increases the elastic modulus of the bio-composites to ranges comparable to the modulus values of mineralized bio-composites. There is evidence of the shearing of collagen molecules between mineral molecules in the gap region, but at lower mineral contents the bending of collagen molecules is also observed. These observations were used to formulate a model to determine the modulus of mineralized bio-composites. The formulated model agrees with the simulations of this study, as well as modulus values determined in experimental studies by others.

For non-mineralized bio-composites at low water contents, the water occupies gap region voids, causing gap region expansion and overlap region contraction. At higher water contents some water also occupies overlap region voids, which reduces the overlap region contraction, and when stress increases, the gap region deforms more than the overlap region. In mineralized bio-composites, the water primarily occupies overlap region voids because of mineral in the gap region, which causes the overlap region to expand while the gap region contracts. As the stress increases, the gap/overlap ratio remains approximately constant. CNTs in non-mineralized bio-composites occupy gap region voids, with the amount and distribution of CNTs and water determining the relative deformation between gap and overlap regions.

The collagen/CNT bio-composites have a much higher heat capacity than the mineralized bio-composites and a lower total bio-composite mass. Overall, collagen/CNT bio-composites have stress vs. strain behaviors, elastic moduli, and gap/overlap ratios comparable to those of mineralized bio-composites, but with a higher heat capacity that would allow collagen/CNT bio-composites to be less susceptible to thermal damage during implantation or functional use in applications like bone machining, as pointed out by Qu and Tomar (Qu and Tomar 2015). This includes uses such as high-speed drilling (Wiggins and Malkin 1976), laser ablation (Nelson et al. 1988), or the curing of cements in implants (Huiskes 1980). The mechanical and thermal characterization of these collagen-based bio-composites is important for designing biocompatible bio-composite materials to treat conditions that affect collagenous tissues via applications such as wound healing, medical implants, drug delivery systems, or prosthetics.

## References

[cit0001] Ahsan AS. 2017. Effect of intrafibrillar mineralization on the mechanical properties of osteogenesis imperfecta bone using a cohesive finite element approach [dissertation]. San Antonio (TX): The University of Texas at San Antonio.

[cit0002] Amirian M, Chakoli AN, Sui JH, Cai W. 2012. Enhanced mechanical and photoluminescence effect of poly (L-lactide) reinforced with functionalized multiwalled carbon nanotubes. Polym Bull. 68:1747–1763.

[cit0003] Balandin AA. 2011. Thermal properties of graphene and nanostructured carbon materials. Nat Mater. 10:569–581. 21778997 10.1038/nmat3064

[cit0004] Boldor D, Gerbo NM, Monroe WT, Palmer JH, Li Z, Biris AS. 2008. Temperature measurement of carbon nanotubes using infrared thermography. Chem Mater. 20:4011–4016.

[cit0005] Charlier J, Lambin P, Ebbesen TW. 1996. Electronic properties of carbon nanotubes with polygonized cross sections. Phys Rev B Condens Matter. 54:R8377–R8380. 9984599 10.1103/physrevb.54.r8377

[cit0006] Currey JD. 2002. Bones: structure and mechanics. Princeton, NJ: Princeton university press.

[cit0007] Depalle B, Qin Z, Shefelbine SJ, Buehler MJ. 2016. Large deformation mechanisms, plasticity, and failure of an individual collagen fibril with different mineral content. J Bone Miner Res. 31:380–390. 26866939 10.1002/jbmr.2705 PMC4915725

[cit0008] Dubey DK, Tomar V. 2009. The effect of tensile and compressive loading on the hierarchical strength of idealized tropocollagen–hydroxyapatite biomaterials as a function of the chemical environment. J Phys Condens Matter. 21:205103. 21825522 10.1088/0953-8984/21/20/205103

[cit0009] Dubey DK, Tomar V. 2013. Ab initio investigation of strain dependent atomistic interactions at two tropocollagen-hydroxyapatite interfaces. J Eng Mater Technol. 135:021015.

[cit0010] Eppell S, Smith B, Kahn H, Ballarini R. 2006. Nano measurements with micro-devices: mechanical properties of hydrated collagen fibrils. J R Soc Interface. 3:117–121. 16849223 10.1098/rsif.2005.0100 PMC1618494

[cit0011] Fielder M, Nair AK. 2018. Effects of hydration and mineralization on the deformation mechanisms of collagen fibrils in bone at the nanoscale. Biomech Model Mechanobiol. 18(1):57–68. 10.1007/s10237-018-1067-y 30088113

[cit0012] Fratzl P, 2008. Collagen: structure and mechanics, an introduction. Collagen: structure and mechanics, 1–13.

[cit0013] Gautieri A, Vesentini S, Redaelli A, Buehler MJ. 2011. Hierarchical structure and nanomechanics of collagen microfibrils from the atomistic scale up. Nano Lett. 11:757–766. 21207932 10.1021/nl103943u

[cit0014] Grant CA, Brockwell DJ, Radford SE, Thomson NH. 2008. Effects of hydration on the mechanical response of individual collagen fibrils. Appl Phys Lett. 92:233902.

[cit0015] Gul-E-Noor F, Singh C, Papaioannou A, Sinha N, Boutis GS. 2015. Behavior of water in collagen and hydroxyapatite sites of cortical bone: fracture, mechanical wear, and load bearing studies. J Phys Chem C. 119:21528–21537. 10.1021/acs.jpcc.5b06285 PMC4675148 26659838

[cit0016] Hamed E, Jasiuk I. 2013. Multiscale damage and strength of lamellar bone modeled by cohesive finite elements. J Mech Behav Biomed. 28:94–110. 10.1016/j.jmbbm.2013.05.025 23973769

[cit0017] Hamed E, Lee Y, Jasiuk I. 2010. Multiscale modeling of elastic properties of cortical bone. Acta Mech. 213:131–154.

[cit0018] Hirata E, Uo M, Takita H, Akasaka T, Watari F, Yokoyama A. 2011. Multiwalled carbon nanotube-coating of 3D collagen scaffolds for bone tissue engineering. Carbon. 49:3284–3291.

[cit0019] Holmes R, Kirk S, Tronci G, Yang X, Wood D. 2017. Influence of telopeptides on the structural and physical properties of polymeric and monomeric acid-soluble type I collagen. Mater Sci Eng C. 77:823–827. 10.1016/j.msec.2017.03.267 28532097

[cit0020] Huiskes R. 1980. Some fundamental aspects of human joint replacement: analyses of stresses and heat conduction in bone-prosthesis structures. Acta Orthop Scand. 51:3–208. 6938104

[cit0021] Ji B, Gao H, Jimmy Hsia K. 2004. How do slender mineral crystals resist buckling in biological materials? Philos Mag Lett. 84:631–641.

[cit0022] Jing Z, Wu Y, Su W, Tian M, Jiang W, Cao L, Zhao L, Zhao Z. 2017. Carbon nanotube reinforced collagen/hydroxyapatite scaffolds improve bone tissue formation in vitro and in vivo. Ann Biomed Eng. 45:2075–2087. 28620768 10.1007/s10439-017-1866-9

[cit0023] Kang Y, Wang Q, Liu YC, Wu T, Chen Q, Guan WJ. 2008. Dynamic mechanism of collagen-like peptide encapsulated into carbon nanotubes. J Phys Chem B. 112:4801–4807. 18366213 10.1021/jp711392g

[cit0024] Karunaratne A, Esapa CR, Hiller J, Boyde A, Head R, Bassett J, Terrill NJ, Williams GR, Brown MA, Croucher PI. 2012. Significant deterioration in nanomechanical quality occurs through incomplete extrafibrillar mineralization in rachitic bone: evidence from in‐situ synchrotron X‐ray scattering and backscattered electron imaging. J Bone Miner Res. 27:876–890. 22161748 10.1002/jbmr.1495

[cit0025] Kemp AD, Harding CC, Cabral WA, Marini JC, Wallace JM. 2012. Effects of tissue hydration on nanoscale structural morphology and mechanics of individual Type I collagen fibrils in the Brtl mouse model of Osteogenesis Imperfecta. J Struct Biol. 180:428–438. 23041293 10.1016/j.jsb.2012.09.012 PMC3685442

[cit0026] Lefèvre E, Guivier-Curien C, Pithioux M, Charrier A. 2013. Determination of mechanical properties of cortical bone using AFM under dry and immersed conditions. Comput Methods Biomech Biomed Engin. 16:337–339. 23923965 10.1080/10255842.2013.815974

[cit0027] Lin L, Samuel J, Zeng X, Wang X. 2017. Contribution of extrafibrillar matrix to the mechanical behavior of bone using a novel cohesive finite element model. J Mech Behav Biomed. 65:224–235. 10.1016/j.jmbbm.2016.08.027 PMC5154908 27592291

[cit0028] MacDonald RA, Laurenzi BF, Viswanathan G, Ajayan PM, Stegemann JP. 2005. Collagen–carbon nanotube composite materials as scaffolds in tissue engineering. J Biomed Mater Res A. 74:489–496. 15973695 10.1002/jbm.a.30386

[cit0029] Meng J, Kong H, Han Z, Wang C, Zhu G, Xie S, Xu H. 2009. Enhancement of nanofibrous scaffold of multiwalled carbon nanotubes/polyurethane composite to the fibroblasts growth and biosynthesis. J Biomed Mater Res A. 88:105–116. 18260129 10.1002/jbm.a.31862

[cit0030] Minary-Jolandan M, Yu M-F. 2009. Nanomechanical heterogeneity in the gap and overlap regions of type I collagen fibrils with implications for bone heterogeneity. Biomacromolecules. 10:2565–2570. 19694448 10.1021/bm900519v

[cit0031] Mueller KH, Trias A, Ray RD. 1966. Bone Density and Composition: age-related and pathological changes in water and mineral content. JBJS. 48:140–148. 5902798

[cit0032] Nair AK, Gautieri A, Buehler MJ. 2014. Role of intrafibrillar collagen mineralization in defining the compressive properties of nascent bone. Biomacromolecules. 15:2494–2500. 24892376 10.1021/bm5003416

[cit0033] Nair AK, Gautieri A, Chang S-W, Buehler MJ. 2013. Molecular mechanics of mineralized collagen fibrils in bone. Nat Commun. 4:1724. 23591891 10.1038/ncomms2720 PMC3644085

[cit0034] Nelson JS, Yow L, Liaw LH, Macleay L, Zavar RB, Orenstein A, Wright WH, Andrews JJ, Berns MW. 1988. Ablation of bone and methacrylate by a prototype mid‐infrared erbium: YAG laser. Lasers Surg Med. 8:494–500. 3230997 10.1002/lsm.1900080508

[cit0035] Nikolov S, Raabe D. 2008. Hierarchical modeling of the elastic properties of bone at submicron scales: the role of extrafibrillar mineralization. Biophys J. 94:4220–4232. 18310256 10.1529/biophysj.107.125567 PMC2480676

[cit0036] Nudelman F, Pieterse K, George A, Bomans PHH, Friedrich H, Brylka LJ, Hilbers PAJ, de With G, Sommerdijk NAJM. 2010. The role of collagen in bone apatite formation in the presence of hydroxyapatite nucleation inhibitors. Nat Mater. 9:1004–1009. 20972429 10.1038/nmat2875 PMC3084378

[cit0037] Nyman JS, Roy A, Shen XM, Acuna RL, Tyler JH, Wang XD. 2006. The influence of water removal on the strength and toughness of cortical bone. J Biomech. 39:931–938. 16488231 10.1016/j.jbiomech.2005.01.012 PMC1941695

[cit0038] Orgel JPRO, Irving TC, Miller A, Wess TJ. 2006. Microfibrillar structure of type I collagen in situ. P Natl Acad Sci USA. 103:9001–9005. 10.1073/pnas.0502718103 PMC1473175 16751282

[cit0039] Padawer G, Beecher N. 1970. On the strength and stiffness of planar reinforced plastic resins. Polym Eng Sci. 10:185–192.

[cit0040] Plimpton S. 1995. Fast parallel algorithms for short-range molecular-dynamics. J Comput Phys. 117:1–19.

[cit0041] Pradhan N, Duan H, Liang J, Iannacchione G. 2009. The specific heat and effective thermal conductivity of composites containing single-wall and multi-wall carbon nanotubes. Nanotechnology. 20:245705. 19471077 10.1088/0957-4484/20/24/245705

[cit0042] Pradhan SM, Katti DR, Katti KS. 2011. Steered molecular dynamics study of mechanical response of full length and short collagen molecules. J Nanomech Micromech. 1:104–110.

[cit0043] Qu T, Tomar V. 2015. Understanding straining induced changes in thermal properties of tropocollagen-hydroxyapatite interfacial configurations. Int J Exp Comput Biomech. 3:62–81.

[cit0044] Rauch F, Glorieux FH. 2004. Osteogenesis imperfecta. Lancet. 363:1377–1385. 15110498 10.1016/S0140-6736(04)16051-0

[cit0045] Samuel J, Park J-S, Almer J, Wang X. 2016. Effect of water on nanomechanics of bone is different between tension and compression. J Mech Behav Biomed. 57:128–138. 10.1016/j.jmbbm.2015.12.001 PMC4798895 26710258

[cit0046] Sasaki N, Odajima S. 1996. Elongation mechanism of collagen fibrils and force-strain relations of tendon at each level of structural hierarchy. J Biomech. 29:1131–1136. 8872269 10.1016/0021-9290(96)00024-3

[cit0047] Saxon SV, Etten MJ, Perkins EA. 2014. Physical change and aging: A guide for the helping professions. New York: Springer Publishing Company.

[cit0048] Silva E, de Vasconcellos LMR, Rodrigues BV, Dos Santos DM, Campana-Filho SP, Marciano FR, Webster TJ, Lobo AO. 2017. PDLLA honeycomb-like scaffolds with a high loading of superhydrophilic graphene/multi-walled carbon nanotubes promote osteoblast in vitro functions and guided in vivo bone regeneration. Mater Sci Eng C. 73:31–39. 10.1016/j.msec.2016.11.075 28183613

[cit0049] Streeter I, de Leeuw NH. 2010. Atomistic modeling of collagen proteins in their fibrillar environment. J Phys Chem B. 114:13263–13270. 20873729 10.1021/jp1059984 PMC3505825

[cit0050] Tan W, Twomey J, Guo D, Madhavan K, Li M. 2010. Evaluation of nanostructural, mechanical, and biological properties of collagen–nanotube composites. IEEE Trans Nanobioscience. 9:111–120. 20215088 10.1109/TNB.2010.2043367

[cit0051] Tanaka M, Sato Y, Zhang M, Haniu H, Okamoto M, Aoki K, Takizawa T, Yoshida K, Sobajima A, Kamanaka T. 2017. In Vitro and In Vivo evaluation of a three-dimensional porous multi-walled carbon nanotube scaffold for bone regeneration. Nanomaterials. 7:46. 28336879 10.3390/nano7020046 PMC5333031

[cit0052] Tang Y, Ballarini R, Buehler MJ, Eppell SJ. 2010. Deformation micromechanisms of collagen fibrils under uniaxial tension. J R Soc Interface. 7:839–850. 19897533 10.1098/rsif.2009.0390 PMC2874230

[cit0053] Thomopoulos S, Marquez JP, Weinberger B, Birman V, Genin GM. 2006. Collagen fiber orientation at the tendon to bone insertion and its influence on stress concentrations. J Biomech. 39:1842–1851. 16024026 10.1016/j.jbiomech.2005.05.021

[cit0054] Timmins PA, Wall JC. 1977. Bone Water. Calc Tiss Res. 23:1–5. 10.1007/BF02012759 890540

[cit0055] van der Rijt JA, van der Werf KO, Bennink ML, Dijkstra PJ, Feijen J. 2006. Micromechanical testing of individual collagen fibrils. Macromol Biosci. 6:697–702. 16967482 10.1002/mabi.200600063

[cit0056] Wang Y, Azais T, Robin M, Vallee A, Catania C, Legriel P, Pehau-Arnaudet G, Babonneau F, Giraud-Guille MM, Nassif N. 2012. The predominant role of collagen in the nucleation, growth, structure and orientation of bone apatite. Nat Mater. 11:724–733. 22751179 10.1038/nmat3362

[cit0057] Wess TJ, Hammersley A, Wess L, Miller A. 1995. Type-I collagen packing, conformation of the triclinic unit-cell. J Mol Biol. 248:487–493. 7537830 10.1016/s0022-2836(95)80065-4

[cit0058] Wiggins K, Malkin S. 1976. Drilling of bone. J Biomech. 9:553–559. 965420 10.1016/0021-9290(76)90095-6

[cit0059] Zanello LP, Zhao B, Hu H, Haddon RC. 2006. Bone cell proliferation on carbon nanotubes. Nano Lett. 6:562–567. 16522063 10.1021/nl051861e

[cit0060] Zhang DJ, Chippada U, Jordan K. 2007. Effect of the structural water on the mechanical properties of collagen-like microfibrils: A molecular dynamics study. Ann Biomed Eng. 35:1216–1230. 17387615 10.1007/s10439-007-9296-8

[cit0061] Zhang P, Huang Y, Geubelle P, Klein P, Hwang K. 2002. The elastic modulus of single-wall carbon nanotubes: a continuum analysis incorporating interatomic potentials. Int J Solids Struct. 39:3893–3906.

